# Rearing Enrichments Affected Ranging Behavior in Free-Range Laying Hens

**DOI:** 10.3389/fvets.2020.00446

**Published:** 2020-08-14

**Authors:** Dana L. M. Campbell, Tim R. Dyall, Jeff A. Downing, Andrew M. Cohen-Barnhouse, Caroline Lee

**Affiliations:** ^1^Agriculture and Food, Commonwealth Scientific and Industrial Research Organisation (CSIRO), Armidale, NSW, Australia; ^2^School of Life and Environmental Science, Faculty of Veterinary Science, University of Sydney, Sydney, NSW, Australia; ^3^School of Environmental and Rural Science, University of New England, Armidale, NSW, Australia

**Keywords:** corticosterone, welfare, individual, chicken, hen, RFID, adaptability, stress

## Abstract

Within Australia, free-range systems are prevalent, but pullets destined for range access are reared indoors. This mismatch between rearing and layer housing may hinder adaptation to the free-range environment. Rearing enrichments could enhance pullet development. A total of 1,386 Hy-Line Brown® chicks were reared inside an experimental facility across 16 weeks with 3 enrichment treatments including (1) a control group with standard floor-housing, (2) a novelty group providing novel objects that changed weekly (“novelty” hens), and (3) a structural group with custom-designed H-shaped structures including opaque sides (“structural” hens). At 16 weeks of age, all pullets were leg-banded with microchips and moved to an experimental free-range system with 9 identical pens (*n* = 3/rearing treatment). From 25 to 64 weeks, individual hen daily ranging behavior was tracked via radio-frequency identification technology and grouped into 6 age periods per rearing treatment. Video footage was used to count the number of hens at different distances on the range for the first 14 days of access, and eggs were assessed for albumen corticosterone concentrations 4 days prior to (*n* = 450) and 1 week after first range access (*n* = 450). Across most age periods, the structural hens spent the most time ranging (*P* ≤ 0.01), the novelty hens showed the fewest number of visits to the range (*P* < 0.0001), and both enriched hen groups had the longest maximum visit durations (*P* ≤ 0.02). Range use increased with age across all treatments with only 3% of hens never going outside. All hens were initially slow to use the range area with fewer novelty hens venturing farther onto the range (*P* ≤ 0.03). The structural hens had higher albumen corticosterone concentrations and variance (both *P* ≤ 0.004) prior to range access. All hens showed an increase in albumen corticosterone following the first week of range access resulting in no differences between rearing treatments in means (*P* = 0.92) and variance (*P* = 0.63). Different enrichments have differing impacts on ranging behavior, but further research is needed to understand the mechanisms of effects, with differences in brain lateralization a potential hypothesis to be tested.

## Introduction

In many countries, the laying hen industry is making a transition away from conventional caged housing toward alternative systems that provide hens with more resources and space to accommodate their behavioral needs. Within Australia, free-range systems are increasingly prevalent as consumers believe these systems provide better hen welfare (1), and eggs are healthier and tastier (2). However, free-range systems provide hens a choice to range or remain indoors, and in some instances, the use of the outdoor range can be low (3). This potentially limits the benefits of this system and/or could reduce consumer satisfaction. There is some evidence that higher use of the range area will improve plumage condition and footpad condition, and reduce toenail length (4, 5). Hens exhibit some important behaviors such as foraging at higher frequencies outdoors than indoors (4, 6).

There are multiple factors affecting hens' use of the range area as adults, both in terms of accessing the range and distribution in the range area (7). These include, for example, the ambient weather (8, 9), shelter on the range (10), additional enrichments on the range (11), and hen age (8, 9, 12). The range area is also a new environment that may require hens to be more adaptable compared with strictly indoor housing systems. Hens that go outside are exposed to weather variation, sunlight, predators, and large temperature fluctuations; typically, the food and water resources necessary for maintaining body condition as well as a high rate of production are located inside. Outdoor access during rearing is also a factor that affects range use as adults (13) but not in all cases (14). For hens that are not reared with outdoor access, there is often a long period (weeks) for hens to become accustomed to the range area following first pop-hole opening (12). It may be stressful to enter the outdoor environment following 16 plus weeks of being inside (first pop-hole opening age varies between commercial producers). Some hens even choose to never exit to the range, and these hens have been identified as more fearful than frequent range users (15–17).

Rearing environments for pullets are important for optimal development, adaptability, and performance as adult hens (18, 19), with studies showing that hens will better adapt to the layer system if they are reared in a similar manner. For example, hens reared in cages will better adapt to a caged layer system following transfer than hens reared in aviaries and placed into cages (20). Producers in Europe that rear free-range pullets with outdoor access report that the management of their rearing flocks to optimize adult performance is a less prominent issue than reported by producers that do not rear outdoors (21). In Australia, pullets destined for free-range systems are typically reared inside due to vaccination schedules and health risks associated with outdoor access, and the logistics of current shed designs, which do not have outdoor ranges. Thus, pullets entering free-range systems may be at a disadvantage, which could impact their range use, health, and welfare as adults. In the absence of feasible outdoor access options, enrichments in the rearing sheds could better prepare pullets for free-range housing. Enrichments can be defined as any addition to the environment that has positive impacts on behavior and/or biology of the animals (22). These can have multiple impacts on the pullets' behavioral, physical, and neurobehavioral development (23). One previous free-range chick enrichment study showed that variable physical and sensory enrichments provided for the first 3 weeks of development improved the hens' adaptation to implemented environmental stressors as adults (12), increased their degree of social flock cohesion (24), but slightly reduced the time spent outside ranging (12). In this previous study, multiple types of stimulation were provided, and thus, it was unclear which aspect (physical, visual, auditory, sensory) may have had the most impact on the pullet's development.

The aim of the current study was to assess the impacts of different types of enrichments provided throughout the rearing period on individual range use of hens across a flock cycle including the use of the length of the range when first provided access and initial stress responses of hens following first pop-hole opening. Two types of enrichments were selected, regularly replaced novel objects to simulate an unpredictable and changing environment, and structures with some opaque sides to allow perching and increased navigation within the pens. It was predicted that both types of enrichments would increase ranging behavior, that the initial range access would require adaptation by the hens to the new environment, and that the novelty hens would be best prepared for this adaptation. This study was part of an overall larger study assessing behavioral and welfare impacts of rearing enrichments in free-range hens.

## Materials and Methods

### Ethical Statement

All research was approved by the University of New England Animal Ethics Committee (AEC17-092).

### Animals and Housing

This study used 1,386 Hy-Line® Brown layers that were reared for 16 weeks in the Rob Cumming Poultry Innovation Centre of the University of New England, Armidale, Australia, and subsequently housed in the Laureldale free-range facility of the University of New England until 65 weeks of age. Day-old chicks were obtained from a commercial supplier (including additional chicks that were not transferred to the laying facility) and placed in 9 floor-litter pens (6.2 m L x 3.2 m W) that were visually isolated via shade cloth hung on the wire pen dividers and distributed across three separate rooms. Each pen had rice hulls as ground litter, round feeders for *ad libitum* access to commercially formulated mash appropriate for different developmental stages, and water nipples. Resources were provided as per the current Australian Model Code of Practice for the Welfare of Animals—Domestic Poultry (25). The pullets were then exposed to three separate rearing enrichment treatments with one replicate of each treatment per room, balanced for location within the room. These included a control group (“control” hens) having no extra materials over the floor litter, a novelty group (“novelty” hens) where novel objects were changed at weekly intervals (e.g., balls, bottles, bricks, brooms, brushes, buckets, containers, pet toys, plastic pipes, strings, water bottles) as well as rotated for location within the room every 3–4 days, and a structural group (“structural” hens) where four custom-designed H-shaped perching structures (L, W, H all 0.60 m) with two solid panels and one open-framed side that could be placed in different orientations were provided for the rearing duration as static enrichment. By 16 weeks of age, bird density was ~15 kg/m^2^ (average 174–190 pullets/pen resulting from chick mortality and some placement error). The temperature and light schedules followed the Hy-Line® Brown alternative management guidelines (26) except that the artificial LED lighting was maintained at 100 lux as the pullets were destined for outdoor access. Rooms were mechanically ventilated as needed, but no cooling system was present. Chicks were infrared beak-trimmed at the hatchery and vaccinated through rearing as per regulatory requirements and standard recommendations for the region.

**Figure 1:**
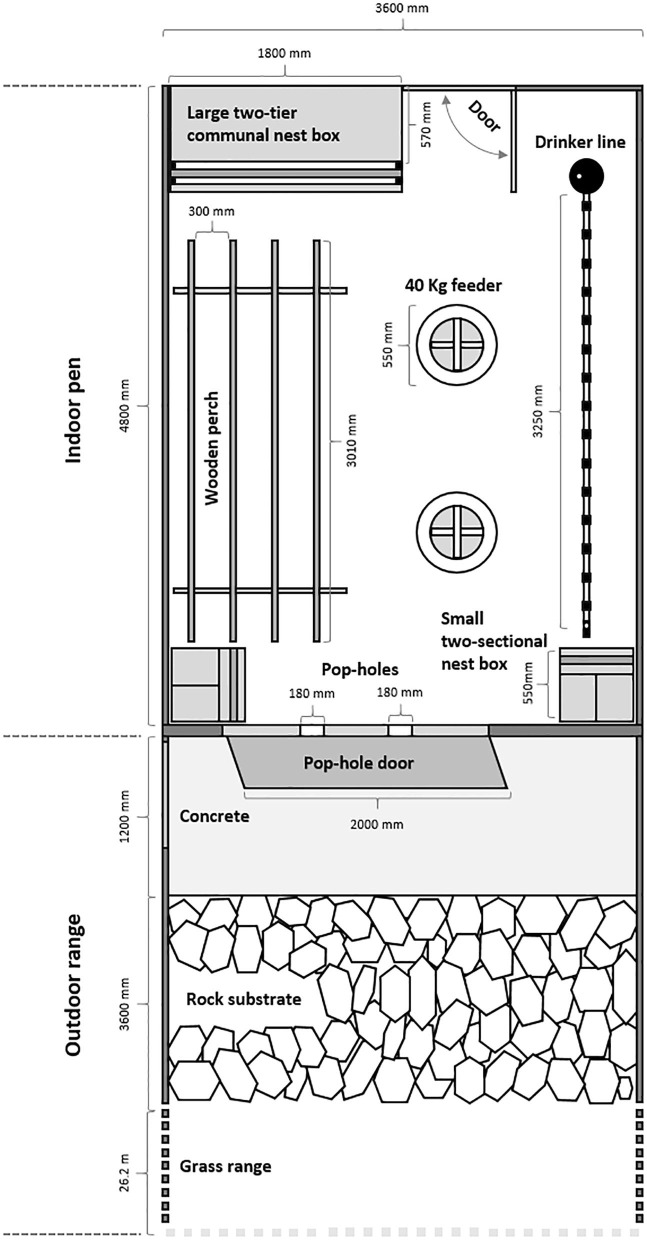
Top-down view of the indoor pen and outdoor range showing placement and dimensions of the indoor perch, nest box, water, and feed resources, the range access pop-holes, and different range substrates. Each of the nine pens had identical indoor configuration except for three pens, which had a radio-frequency identification box in the front right corner that the small nest box sat upon (the small nest boxes were elevated by cinder blocks in the remaining pens). Reproduced from (27).

At the end of rearing, 16-week-old pullets were transferred to the Laureldale free-range facility and socially remixed within pen replicates of their rearing treatment across 9 pens within a single shed (3 pen replicates per rearing treatment of similar group sizes to the rearing period). The indoor pens were of the same configuration (Figure 1) and visually isolated via shade cloth. Each pen contained nest boxes, perches, feeders, and water nipples to fulfill the requirements of the Australian Model Code of Practice for the Welfare of Animals—Domestic Poultry (25). Perching space was 10 cm per bird due to logistical space restrictions within the pen, but hens also perched on the tops of the feeders and waterlines. Rice hulls were placed on the floor with regular raking management and one complete litter replacement midway through the flock cycle. The LED lighting schedule gradually increased to 16 h light and 8 h dark by 30 weeks of age with an average pen light intensity of 10.0 (± 0.84 SE) lux (Lutron Light Meter, LX-112850; Lutron Electronic Enterprise CO., Ltd, Taipei, Taiwan) as measured at birds' eye height from three pen locations (front, middle, back) when the pop-holes were closed. This light intensity was the highest that could be achieved with the shed lighting system. The shed was fan-ventilated with no temperature or humidity control.

The nine indoor pens were each connected to an outdoor range area (Figure 1) accessible via two pop-hole openings (18 cm W × 36 cm H) and visually isolated from each other via shade cloth on the wire fences. Automatic pop-holes were first opened at 25 weeks of age (May 2018) allowing daily access to the hens for most of the daytime. The pop-holes opened at 9:15 am and closed after sunset daily. This equated to ~9 h of available ranging time across winter followed by ~11 h of available ranging time after daylight saving time started (October 2018 until trial completion in January 2019). The range area comprised a concrete path, followed by river rock and then a grassed area devoid of trees or additional shelters (Figure 1). Visual estimation from photos showed that the ranges were initially 90% covered in grass. By 8 weeks after the first range access, the grass was either destroyed by the hens or had gone through winter die-out. There was some grass regrowth in the spring (6 months after first range access) with up to 40% coverage in some pens (3 pens 0%: 1 of each treatment, 4 pens 20%: 2 novelty, 2 structural, 2 pens 40%: 2 control), but by summer (8 months after the first range access), there were only hen-resistant weeds scattered in the bare dirt. A temperature logger (Tinytag Plus 2, TGP-4500; Gemini Data Loggers Ltd, West Sussex, UK) was placed out on the range to record average daily temperature throughout the flock cycle (hourly measurements were recorded).

### Radio-Frequency Identification (RFID) System and Data

Before transfer to the laying facility, all hens were banded with microchips [Trovan® Unique ID 100 (FDX-A): operating frequency 128 kHz] glued into adjustable leg bands (Roxan Developments Ltd, Selkirk, Scotland). Radio-frequency identification (RFID) systems were set up in the indoor pens (as per (28)). These systems were designed and supported by Microchips Australia Pty Ltd (Keysborough, VIC, Australia) with equipment developed and manufactured by Dorset Identification B.V. (Aalten, the Netherlands) using Trovan® technology. Antennas were placed within the two pop-holes per pen that allowed range access, and the movement of individual hens out to the range and back inside to the pen was tracked. The RFID system recorded the date and time of each banded bird passing through the pop-hole and in which direction (onto the range, or into the pen) with a precision of 0.024 s (maximum detection velocity 9.3 m/s). Individual ranging data were collected daily from 25 until 64 weeks of age.

These daily RFID data from individual hens (272 days) were grouped into six time periods comprising 25–27, 27–31, 31–38, 38–44, 47–54, and 55–64 weeks of age. Due to technical malfunction, unforeseen circumstances, and experimental interventions (e.g., weighing days and a stressor period as part of a separate dataset), some days of data were excluded resulting in a total of 232 days analyzed across the 272-day recording period. There were 6 days of data missing for one control and one novelty pen within the 27–31 weeks recording period due to technical malfunction. Once grouped, the data were run through a custom-designed software program written in the “Delphi” language (Bryce Little, CSIRO, Agriculture and Food, St Lucia, QLD, Australia) that filtered out any unpaired or “false” readings that may occur if, for example, a hen sits inside the pop hole but does not complete a full transition onto the range or back into the pen. The same program summarized the daily data to provide an average of hours outside, the number of visits outside, the maximum individual visit time, and the total percentage of available days accessed per individual hen per age period.

### Video Recording and Data Collection

Nine Hikvision Network cameras (Model DS-2CD2232-I5 4 mm, Hikvision, Hangzhou, China) were installed to capture the range area of each pen (one camera per pen) across 14 days during pop-hole opening times excluding ~1.2 m in front of the pop holes (due to the camera angle). Video recordings were later decoded by a single observer (blind to rearing treatment) who counted the number of hens present at different distances from the shed across the length of the range area (1.2–5, 5–10, 10–20, and 20–31 m) every 30 min for the first 2 weeks of range access (total 14 days).

### Albumen Sampling

At 24 weeks of age, 4 days before the hens were provided outdoor access for the first time, a total of 50 eggs from each pen were randomly selected in the morning across all laying locations (floor, small, and large nest boxes). Substantially dirty eggs were not included. The same number of eggs was collected again 7 days following initial range access. On the day of collection, all eggs were weighed and broken open; the albumen was separated from the yolk then weighed and stored at −20°C until analysis via radioimmunoassay following the procedures reported in Downing and Bryden (29). All albumen corticosterone analyses were conducted blind to rearing treatment.

### Data and Statistical Analyses

All analyses were conducted in JMP14.0 (SAS Institute, Cary, NC, USA) with α set at 0.05. Data were checked for normality and homoscedasticity by visual inspection of the model residuals; data transformations or non-parametric tests were applied where necessary. It was assumed that data from individual pens were independent from each other due to physical and visual separation. The video data were averaged per day per pen for each measured distance (14 days × 9 pens × 4 distances = 504 data points). Mean count values were square-root-transformed and analyzed using separate general linear mixed models (GLMMs) per distance with the fixed effects of day, rearing treatment, and their interaction and pen nested within rearing treatment included as a random effect.

The albumen corticosterone data were collated per individual sample within each treatment for prior to and after the first range access (*n* = 900). Data could not be transformed to meet assumptions of homogeneity of variance, so Kruskall–Wallis tests were applied to assess for differences between group means prior to and following range access (pen was not able to be included as a blocked effect due to unequal sample sizes). Levene's tests were applied to assess for differences in variance between treatment groups both prior to and following range access.

The RFID data of mean daily time outside (h), the mean daily number of range visits, the mean maximum individual visit time (h), and the mean proportion of available days the range was accessed were compiled per individual hen across three rearing treatments and six age periods. There was one datapoint per hen within each age period for those hens that used the range (this number increased across time as more hens started ranging). While ranging of individual hens within a pen may have been affected by other hens, we included data at the hen level as individual birds were able to be tracked. Data could not be transformed to meet assumptions of homogeneity of variance, so Kruskall–Wallis tests were applied to assess for differences between treatment groups separately for each age period. Pen was unable to be included as a blocked variable due to unequal sample sizes. Individual-bird data for an entire time period were excluded if that bird died within that specific time period. Across the flock cycle, 29 hens (2.1%) died or were removed for poor health reasons. The hours outside, the number of visits, and the proportion of available days the range was accessed were compared using Spearman's rank correlations between each successive age period, and between the first and last age periods separately for each rearing treatment. Finally, all hens that had no visits recorded on the range across the last age period were selected, and their proportion of days accessed across all previous age periods were graphed to display consistency for the most extreme indoor hens. Raw data are presented in the figures.

## Results

**Figure 2:**
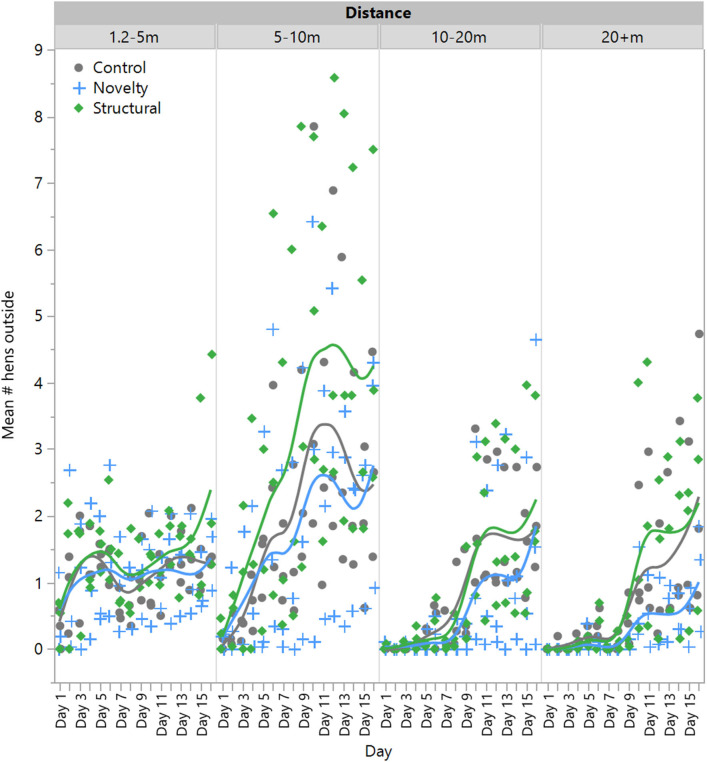
The mean number of laying hens from three rearing enrichment treatments (control, novelty, structural) outside at increasing distances of the range length across the first 14 days of range access. Individual data points indicate daily means for each pen per rearing treatment.

There was no significant effect of rearing treatment on the number of hens outside at 1.2–5 m [*F*_(2,6)_ = 0.23, *P* = 0.80] or 5–10 m [*F*_(2,6)_ = 0.43, *P* = 0.67], but there was a significant effect of day [1.2–5 m: *F*_(1,132)_ = 31.54, *P* < 0.0001; 5–10 m: *F*_(1,132)_ = 220.11, *P* < 0.0001] with range use increasing across time (Figure 2). There was no significant interaction between rearing treatment and day [1.2–5 m: *F*_(1,132)_ = 0.70, *P* = 0.50; 5–10 m: *F*_(1,132)_ = 2.35, *P* < 0.10]. However, there was a significant interaction between rearing treatment and day for hens at 10–20 m [*F*_(2,132)_ = 3.78, *P* = 0.03] and 20+ m [*F*_(2,132)_ = 5.70, *P* = 0.004], with novelty hens showing a comparatively lower increase in the use of these farther distances across time (Figure 2). All hens did increase their range use across the 2-week period at these farther distances [10–20 m: *F*_(1,132)_ = 318.56, *P* < 0.0001]; 20+ m: *F*_(1,132)_ = 273.38, *P* < 0.0001, Figure 2.

**Figure 3:**
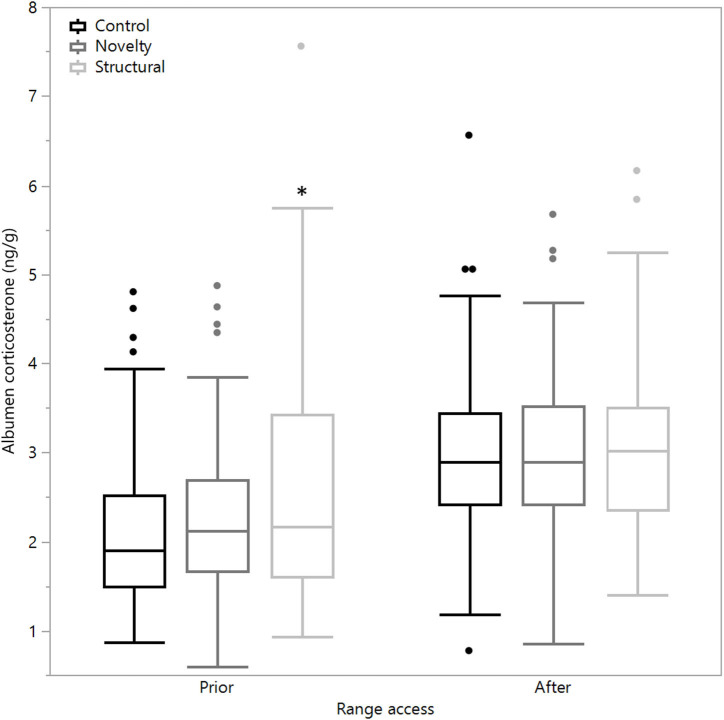
The mean corticosterone concentrations (ng/g) of egg albumen from hens exposed to three rearing enrichment treatments (control, novelty, structural) sampled 4 days prior to and 7 days after the first range access. Box ends represent the first and third quartiles with whiskers extending to data within 1.5 x the interquartile range or upper and lower data points (excluding outliers) if the data do not reach the computed ranges. Isolated data points indicate outliers. The asterisk indicates that the structural hens significantly differed from the other treatment groups prior to the range access.

There was a significant difference between rearing treatments prior to range access in the concentrations of albumen corticosterone (χ^2^ = 11.03, df = 2, *P* = 0.004) with the structural hens showing a higher corticosterone concentration as well as significantly higher variance [*F*_(2,447)_ = 23.12, *P* < 0.0001, Figure 3]. However, following range access, there were no differences between treatment groups in means (χ^2^ = 0.18, df = 2, *P* = 0.92) or variance [*F*_(2, 296.33)_ = 0.46, *P* = 0.63], but all treatment groups showed elevated concentrations (Figure 3).

**Figure 4:**
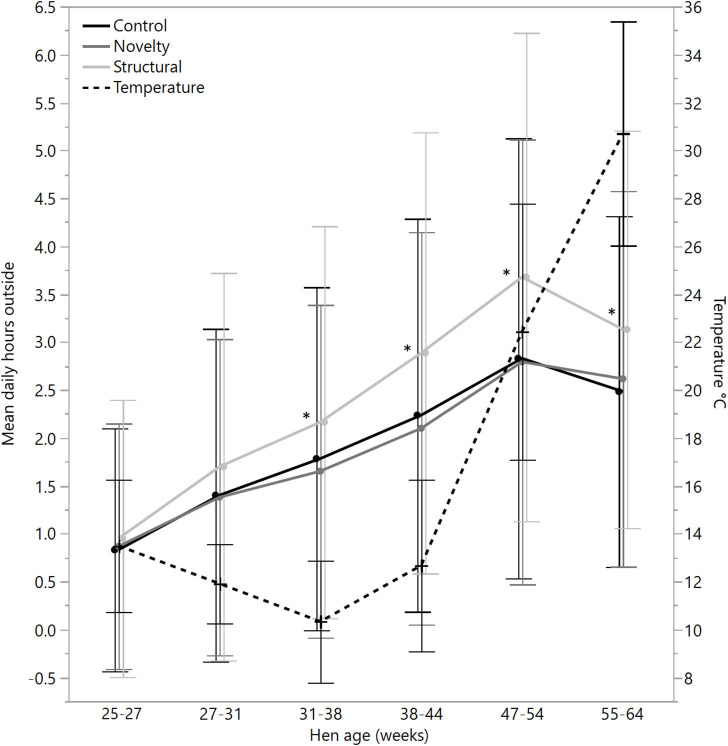
The mean (± SD) daily hours spent outside on the range for hens from three rearing enrichment treatments (control, novelty, structural) across hen age periods. The mean daily temperature during ranging hours is also plotted. Asterisks indicate that the structural hens differed significantly from the control and novelty hens across four of the six age periods.

**Figure 5:**
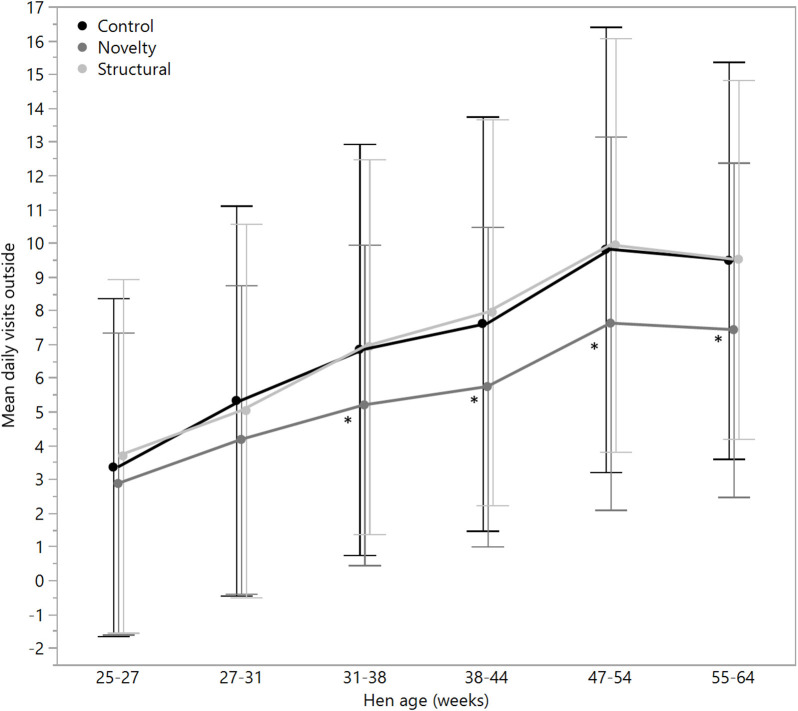
The mean (± SD) daily visits to the outside range for hens from three rearing enrichment treatments (control, novelty, structural) across the periods of hen age (weeks). Asterisks indicate that the novelty hens differed significantly from the control and structural hens across four of the six age periods.

**Figure 6:**
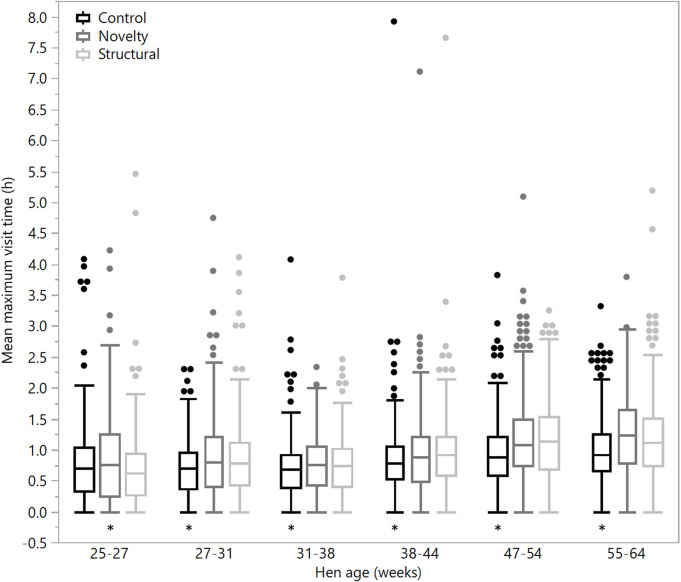
The mean maximum daily visit time outside for hens from three rearing enrichment treatments (control, novelty, structural) across the periods of hen age (weeks). Box ends represent the first and third quartiles with whiskers extending to data within 1.5 x the interquartile range or upper and lower data points (excluding outliers) if the data do not reach the computed ranges. Isolated data points indicate outliers. Asterisks indicate that the control hens differed from both enriched treatment groups across five of the six age periods.

There were no significant differences between rearing treatments in the daily hours outside across the first two age points (25–27 weeks: χ^2^ = 1.13, df = 2, *P* = 0.57; 27–31 weeks: χ^2^ = 2.15, df = 2, *P* = 0.34), but there were significant differences between rearing treatments for each age period for the remainder of the flock cycle (χ^2^ = 12.46–34.27, df = 2, *P* ≤ 0.002) with the hens from the structural rearing treatment spending the most time outside (Figure 4). There were no significant differences between rearing treatments in the number of daily visits to the range at 25–27 (χ^2^ = 1.11, df = 2, *P* = 0.57) and 27–31 weeks of age (χ^2^ = 2.84, df = 2, *P* = 0.24). For the remaining time periods, there were significant differences between rearing treatments (χ^2^ = 22.44–47.20, df = 2, *P* < 0.0001) with the novelty hens showing the fewest visits (Figure 5). There were significant differences between rearing treatments for the maximum visit duration across all ages (χ^2^ = 6.37–54.99, df = 2, *P* ≤ 0.04), with generally the enriched hens (novelty and structural) both showing longer maximum visit times than the control hens (Figure 6).

**Figure 7:**
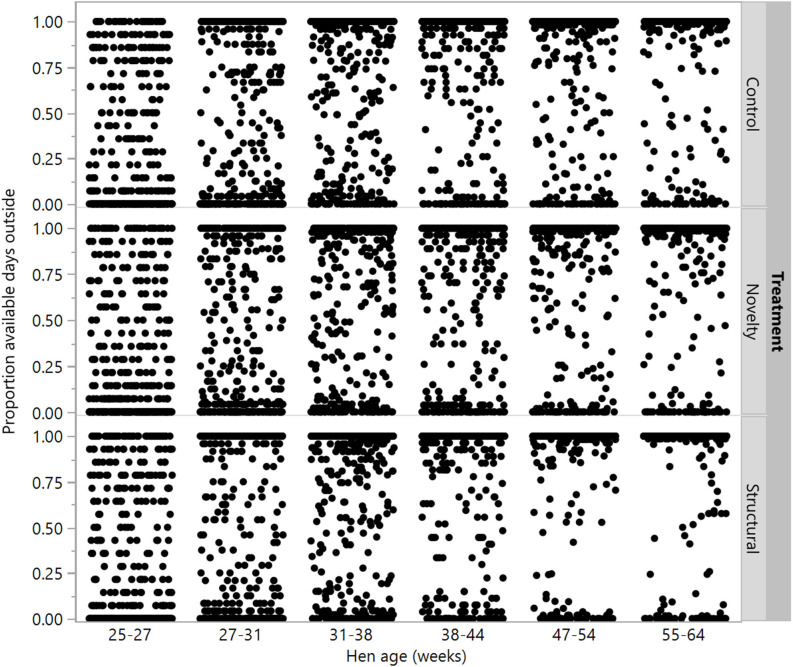
The proportion of available ranging days that individual hens from three rearing treatments (control, novelty, structural) went outside across the flock cycle (25–64 weeks). Differences between treatments were found at 38–44, 47–54, and 55–64 weeks of age.

There were no differences between rearing treatments in the proportion of available days that individual hens went outside at 25–27, 27–31, and 31–38 weeks (χ^2^ = 0.02–4.49, df = 2, *P* ≤ 0.99), but there were differences between groups at the remaining age points (χ^2^ = 14.40–23.63, df = 2, *P* ≤ 0.0007) with the structural hens spending the most days outside (Figure 7).

**Figure 8:**
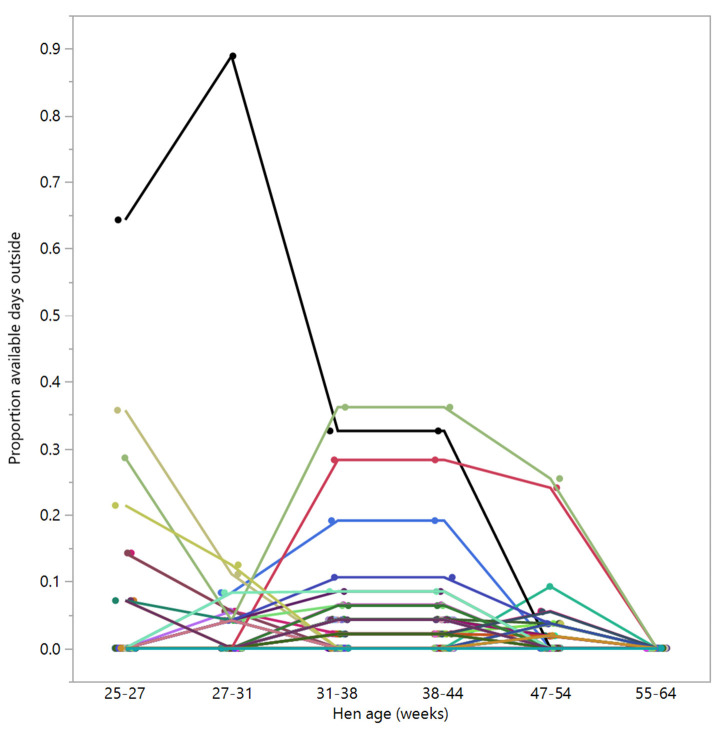
The proportion of available days that the hens (*n* = 98) spent outside across different age periods. Displayed hens were selected based on showing no days outside in the last age period (55–64 weeks). Different colors represent individual hens.

There were 98 hens that were registered with zero days outside across 55–64 weeks of age; of these, 39 hens were registered as never going outside at any point across the trial duration (control: *n* = 13, novelty: *n* = 16, structural: *n* = 10), and the remaining hens did go outside sometimes but for consistently low proportions of time (Figure 8).

There were correlations of 0.74 to 0.95 across adjacent age periods for daily hours, daily visits, and proportion of days outside (Table 1). However, there were lower correlations (0.30–0.46) for the first and last measured age periods (Table 1).

**Table 1:** The ρ values for Spearman's rank correlations between adjacent hen age periods for mean daily ranging hours, visits, and proportion of days spent outside for hens from three rearing enrichment treatments (control, novelty, structural).

	**Rearing treatment**		
**Age (weeks)**	**Control**	**Novelty**	**Structural**
	**Ranging hours**^**a**^		
25–27 and 27–31	ρ = 0.74	ρ = 0.74	ρ = 0.80
27–31 and 31–38	ρ = 0.82	ρ = 0.81	ρ = 0.81
31–38 and 38–44	ρ = 0.92	ρ = 0.91	ρ = 0.92
38–44 and 47–54	ρ = 0.92	ρ = 0.90	ρ = 0.92
47–54 and 55–64	ρ = 0.94	ρ = 0.95	ρ = 0.95
*25–27 and 55–64*	*ρ = 0.38*	*ρ = 0.36*	*ρ = 0.46*
	**Ranging visits**^**a**^		
25–27 and 27–31	ρ = 0.75	ρ = 0.76	ρ = 0.80
27–31 and 31–38	ρ = 0.84	ρ = 0.81	ρ = 0.81
31–38 and 38–44	ρ = 0.93	ρ = 0.89	ρ = 0.89
38–44 and 47–54	ρ = 0.88	ρ = 0.87	ρ = 0.88
47–54 and 55–64	ρ = 0.90	ρ = 0.90	ρ = 0.88
*25–27 and 55–64*	*ρ = 0.37*	*ρ = 0.33*	*ρ = 0.46*
	**Proportion days ranging**^**a**^		
25–27 and 27–31	ρ = 0.81	ρ = 0.77	ρ = 0.83
27–31 and 31–38	ρ = 0.84	ρ = 0.82	ρ = 0.86
31–38 and 38–44	ρ = 0.81	ρ= 0.82	ρ = 0.83
38–44 and 47–54	ρ = 0.84	ρ = 0.85	ρ = 0.74
47–54 and 55–64	ρ = 0.79	ρ = 0.80	ρ = 0.80
*25–27 and 55–64*	*ρ = 0.34*	*ρ = 0.31*	*ρ = 0.30*

*A comparison between the first and last age periods for each ranging variable is italicized*.

a *All P < 0.0001*.

## Discussion

This study assessed the impacts of different rearing enrichments on subsequent range use by adult hens in an experimental setting across a production cycle. Hens that were provided with perching structures including opaque sides spent the most time on the range, and hens that were exposed to different novel objects showed fewer visits to the range; both these enriched treatments typically supported longer individual range visit times than the control hens. There were individual differences between hens in how often they accessed the range across all rearing treatments with an increase in range use as hens aged. Most hens showed some range use by the end of the flock cycle, but a small proportion remained inside across the trial duration. Hens were slow to first use the range and showed elevated albumen corticosterone concentrations at the end of the first week. These results indicate that enrichments for pullets reared indoors can modify subsequent range use with impacts across the flock cycle.

Two types of enrichments were tested in this study that had disparate, yet sustained impacts on ranging. In a previous study that applied enrichments for the first 3 weeks of life (12), multiple types of enrichments (stimulatory and physical) were combined. These enrichments resulted in a small reduction in hours outside for the enriched hens and reduced corticosterone responses to implemented stressors. However, it was uncertain specifically what aspect of the enrichments may have had the greatest effect. The increase in ranging hours by the structural hens in the current study may have been due to improvements in their spatial navigation abilities. Previous research has shown some effects of elevated structures during rearing on the speed of completing cognitive tasks in chicks (30) or spatial jumping tasks in pullets (31). Laying hens reared in aviaries also showed improved three-dimensional use of their new pens when transferred to the laying facility compared with hens reared in cages, although these differences were not sustained past the first 4 weeks following transfer to the laying facility (32). Chicks with exposure to occlusion barriers within the first 2 weeks of development showed some modification of their spatial behavior compared with control chicks receiving no occlusion experience (33). The structural groups had experience with large opaque barriers throughout rearing, although some of the initial objects in the novelty group (cinder blocks, buckets) may have also functioned as occlusion barriers to the small chicks. The structural hens may have felt more competent in moving between the indoor and outdoor areas, thus increasing the overall amount of time they spent outdoors. However, contrary to predictions of improved spatial abilities in the structural hens, the novelty hens showed the greatest perching within the home pen upon first transfer to the layer facility at 16–17 weeks of age (34) and continued to show the highest use of the large two-tiered nest boxes (compared with small ground nest boxes or floor-laying) across the production cycle (27). The novelty hens adapted to the home pen more rapidly, which may have led to the increased time spent inside rather than out on the range once the pop-holes were opened. Finally, the structural hens may have also spent more time on the range as a result of improved social interactions. The range area would have a reduced stocking density (even at maximum occupancy) compared with the indoor pen, and more hens outside consequently would lower the indoor stocking density. Perhaps the ability to perch or move out of sight of conspecifics during rearing improved the mediation of conspecific interactions, thus improving their social spacing as adults—a hypothesis that remains to be tested.

Results indicated that in the initial period of range access, hens probably experienced a level of stress associated with exposure to a new, unfamiliar housing environment, which could account for the elevated albumen corticosterone and their hesitation to venture outside and/or use the full range area. This was anticipated given previous findings of low range use initially (12, 14) and the expectation that the outdoor environment was highly novel following a long period of indoor-only exposure. Additionally, the hens were quite old at age of first access (25 weeks), an intentional experimental decision as hens might be less adaptable at the older age, thus increasing the testing stringency of any rearing enrichment effect. Alternatively, hens may have been aroused with the new experiences available to them; future tests combining valence with arousal measures would confirm the effects of initial pop-hole opening on hen affect. Contrary to expectations, the novelty hens were slowest to start using the range and travel along its full length away from the shed. The structural hens showed the smallest change in albumen corticosterone concentrations between baseline and following range access suggesting that they could have been less stressed and more capable of adapting to the range more readily. However, their mean corticosterone concentrations and variance were higher in the baseline samples compared with the other rearing treatment groups, and it is unclear why this may have been. The assay used to determine the corticosterone concentrations is a radioimmunoassay and uses antiserum that has some cross-reactivities to other steroids (29). A recent HPLC-MS-MS analysis of egg albumen reported that the corticosterone concentrations are low (35), but there was little background information provided for the hens used in the study. Comparisons of mean percentage egg production between treatment groups across 7 days prior to the baseline sampling showed some differences between the control and both enriched treatment groups (control: 87.5% production; novelty: 93.5%; structural: 93.1%), similarly across 7 days prior to the second sampling point (control: 88.3%; novelty: 91.7%; structural: 90.0%), so it is unclear to what degree cross-reactivities may have affected the corticosterone results. Other physiological measures such as blood profiles instead of or in addition to albumen corticosterone may be more informative but are difficult to measure due to the stress of handling the birds. Following the first 6 weeks of ranging, the novelty hens spent a similar amount of time on the range as the control hens, but they had fewer visits, with longer maximum durations, similar to those of the structural hens. Thus, both enrichment treatments had effects on the hens' behavior, but the mechanism of their impact is unclear. It is possible that the enrichments resulted in different degrees of brain laterality and hemispheric dominance in the hens.

A lateralized brain will improve the ability to respond to concurrent stimuli (e.g., searching for food while under threat from a predator) (36) and likely has implications for animal welfare such as the display of a negative bias or an elevated response to stressful situations (37, 38). The left hemisphere controls established behavioral patterns compared with the right hemisphere that attends to unexpected stimuli; an overview of the hemispheric specializations is provided in Rogers (36) and Rogers and Kaplan (38). The different types of enrichments provided may have either improved the degree of hemispheric flexibility and how the hens react to stimuli and their surrounding environment (38), or increased the dominance of a specific hemisphere thus altering the main hemisphere attending to the environment. Changes in cellular neural processes following enrichment have been demonstrated in rodents including differences based on the period of exposure (39). Thus, confirmation of the impacts of different types of enrichments on laterality and neural pathways warrants further investigation, particularly the optimal timing of enrichment exposure in pullets (23).

The number of hens outside and the time spent outside generally increased with age indicating acclimation to the range area, but there was a drop at the end of the production cycle, which may have resulted from the increasing summer temperatures (see Figure 4). Across all rearing treatments, there were clear individual differences in the degree of time hens spent ranging, which is further confirmation to the findings of multiple previous studies [e.g., (9, 12, 28, 40)]. This individual variation was present across all rearing treatments indicating that no specific rearing environment eliminated variability in ranging patterns between hens. The effectiveness of provided enrichments could be impacted by the degree of interaction that each hen specifically had with the enrichment objects and/or their perception of them (i.e., stimulating, stressful, benign). The correlations of range use between successive age periods indicate consistency in individual ranging patterns, which was found to have implications for some welfare measures of the hens in this study, although most hens were in relatively good condition at the end of the trial (41). Across the whole trial, ~3% of the hens never went outside. These likely represent an extreme end of the population distribution and may be related to differences in affective states with more fear and anxiety in some hens leading them to remain indoors within a free-range system (16). A free-range system that provides a choice of different environments may thus be conducive to catering for individual differences in welfare needs (42).

## Conclusion

Providing range access during rearing may improve range access as adults, but this is not a feasible strategy across all countries. Rearing enrichments may be an alternative to improve an adult hen's use of the range. Different types of enrichments can have varying impacts on ranging behavior, where in the current study, stable perching structures with opaque sides provided during rearing led to the highest use of the range area in adult hens. The mechanism of impact may have been through changes in brain lateralization, but further studies would be needed to test this hypothesis.

## Data Availability Statement

The datasets generated for this study are available on request to the corresponding author.

## Author Contributions

DC and CL contributed to the conception and design of the study. DC, TD, JD, and AC-B conducted the experiments. DC performed the statistical analyses and wrote the first draft of the manuscript. DC, JD, and CL revised the manuscript. DC and CL acquired funding for the research. All authors contributed to manuscript revision, read and approved the submitted version.

## Conflict of Interest

The authors declare that the research was conducted in the absence of any commercial or financial relationships that could be construed as a potential conflict of interest.
